# A Real Rip Van Winkle–The Strange Case of Dr. Bruno

**Published:** 1913-11

**Authors:** 


### Abstracts and Selections.

### A Real Rip Van Winkle—The Strange Case of Dr. Bruno.

The editor of the *Texas Medical Journal*, Dr. Daniel, has written one of the most fascinating books of the century. The book is remarkable, not only as a love story in which the talented author describes most vividly the vibrations of the *chordae tendinae,* but he also delves deeply into the science of the phenomena of life—biology, cytology, physiological chemistry, electro-dynamics, psychology, amnesia (double personality) and suspended animation. Never was there written a more beautiful, weird, scientific, tragic love story. It pulses with manly, chaste, soul-stirring events of intense human and scientific interest.

From Literary Department, *Pacific Medical Journal*, by Winslow Anderson, A. M., M. D., M. R. C. P., London; M. R. C. S., England; L. S. A., London, etc.; Professor of Gynecology and Abdominal Surgery in the College of Physicians and Surgeons of San Francisco, etc., Editor-in-Chief.

Von Boeckmann-Jones Company, Austin, Texas. Price, $1.50, or with the "Red Back" one year $2.00.

